# Perceptions about medication management transitioning from hospital to home among older adults with coronary artery disease: a longitudinal qualitative study

**DOI:** 10.1186/s12877-026-07610-8

**Published:** 2026-05-12

**Authors:** Mengqi Xu, Suzanne Hoi Shan Lo, Lingyan Zhu, Xiaoli Huang

**Affiliations:** 1 The Nethersole School of Nursing, Faculty of Medicine, Chung Chi College, The Chinese University of Hong Kong, 8/F, Esther Lee Building, Shatin, New Territories, Hong Kong SAR China; 2 Department of Nursing, Shanghai Sixth People’s Hospital Affiliated to Shanghai Jiao Tong University School of Medicine, Shanghai, China

**Keywords:** Coronary artery disease, Older adults, Medication management, Transition to home, Qualitative study

## Abstract

**Background:**

Returning home from the hospital is a critical timepoint for medication management among older adults (adults aged 60 years or over) with coronary artery disease. Older adults experience changes in their medication regimens, and a shift from supervised medication management to self-management, leading to a higher risk of medication discrepancies. However, limited studies are available to understand the changes in their perceptions about medication management. This study aims to explore the changes in perceptions about medication management among older adults with coronary artery disease transitioning from hospital to home.

**Methods:**

A longitudinal qualitative study was conducted with a purposive sample of older adults with coronary artery disease (*n* = 28) and caregivers (*n* = 10), during June to September 2024. Older adults participated in interviews before and one month after hospital discharge to home. Semi-structured interviews were conducted regarding the older adults’ and caregivers’ perceptions of medication management. Data was analysed using thematic and framework analysis.

**Results:**

Two themes along with five sub-themes were developed, including: (1) Theme 1. From compliance to engagement in medication management: Sub-theme (1) Feeling compelled to accept to take the medications by disease, Sub-theme (2) Leaving health to the healthcare professionals; (2) Theme 2. Struggling in balancing the benefits and risks of medications: Sub-theme (1) Evolving understanding about the benefits of medications, Sub-theme (2) Predominant concerns about adverse drug events, Sub-theme (3) Indecisiveness in maintaining adherence. The views of older adults and caregivers were consistent across the themes.

**Conclusion:**

Changes in perceptions about roles, disease and medications were observed among older adults with coronary artery disease transitioning from hospital to home. Older adults simply followed instructions, but shifted to independent administrators after discharge. They accepted to take medications after discharge, as perceiving consistent threat of coronary artery disease. They had evolving understanding in the benefits and risks of medications and struggled in balancing them. More patient-centered and culturally sensitive interventions are warranted for future research. Providing continuous support in medication management, developing positive perceptions of mild side effects and skills in addressing severe side effects, and involving caregivers are important strategies to optimise medication management.

**Supplementary Information:**

The online version contains supplementary material available at 10.1186/s12877-026-07610-8.

## Introduction

Coronary artery disease (CAD) is the leading cause of death worldwide [1]. In China, the incidence of CAD rose from 177.1 per 100,000 person year in 1990 to 197.4 per 100,000 person year in 2019 [2]. It is estimated that over two-thirds of CAD deaths are of older adults in China [3].

Medication treatment is the priority of CAD secondary prevention, including renin-angiotensin system inhibitors, beta-blockers, lipid-lowering drugs and antiplatelet agents [4, 5]. Medication management is a comprehensive process of prescribing safe and effective regimens and ensuring adherence of the patients to the regimens [6]. Prescribing, dispensing, administering, and monitoring are crucial steps for ensuring medication safety [7]. During hospitalisation, the healthcare professionals manage the medications, and older adults follow their orders. After discharge, the self-administration starts and older adults are responsible for their medication management. Compared with simply adhering to medications, it requires more about understanding of the medications, support from caregivers and communication with healthcare professionals [8]. Ageing puts a huge burden on CAD prevention. Older adults encountered significant challenges in medication management, such as fear of side effects, polypharmacy and the lack of medication management capacity. Older adults have a high risk of side-effects of CAD medications, such as haemorrhage, myopathy and hepatotoxicity, because of the decreased liver, and kidney functions [9–11]. Approximately 96.34% of older adults with CAD were exposed to polypharmacy, which increased the burden in medication management [12]. Moreover, 55.9% of older adults with CAD reported having inadequate skills in taking medications on time and 57.5% of them received reminders from others to avoid forgetting to take the medications [13].

Ensuring medication safety is one of the key challenges in the transition of care worldwide [14]. Returning home from the hospital is a critical timepoint for medication management among older adults. A cross-sectional study found that the discrepancies between the prescriptions and medications taken happened in 55.4% of middle and old aged adults with CAD within one to two months after discharge in China [15]. Among the discrepancies, 12.4% were caused by feeling unnecessary to take the medications when asymptomatic, 5.8% resulted from fear of side effects and 2.5% were due to distrusting the benefits of the medications. Several longitudinal studies explored the changes in medication adherence among patients with CAD after hospital discharge. Lu et al. identified three patterns among patients with CAD within three months of discharge, including sustaining/declining to nonadherence, improving adherence, and maintaining adherence [16]. Absence of stents and more disease knowledge were associated with medication nonadherence. In another study, irregular users, early gaps, fast-declining, and slow-declining adherence were observed in three months of discharge among patients with CAD [17]. Therefore, medication management behaviours in the transition from hospital to home are dynamic, but limited evidence is available to understand the changes in older adults’ perceptions about medication management.

Caregivers support the older adults with CAD and interact with them in medication management at home, whose views may interconnect and supplement the perceptions of older adults. Especially in China, people usually live with family members because of filial piety [18]. Caregivers contribute to medication management by obtaining and distributing medications, reminding the patients about medication taking, and monitoring the effects [19, 20]. The negative perceptions of CAD medications by caregivers may lead to nonadherence among the patients [21].

This study explored the changes in perceptions of medication management among older adults with CAD when transiting from hospital to home. Additionally, the perceptions of caregivers were explored to supplement the perceptions of the older adults about medication management.

## Methods

### Design

This is a longitudinal qualitative study, including older adults with CAD and caregivers. Due to the dynamic nature of medication management behaviours, a longitudinal qualitative study approach was chosen to identify the changes in perceptions about medication management across the transition [22]. Consolidated Criteria for Reporting Qualitative Research (COREQ) checklist guided reporting of the methods and results (Supplementary 1).

### Ethical consideration

Declaration of Helsinki was followed. Approval for the study protocol was obtained from the Survey and Behavioural Research Ethics Committee of The Chinese University of Hong Kong (SBRE-23-0688) and the study setting (2024-074-(1)). Written informed consent for publication was obtained from all participants before commencing interviews.

### Setting

This study was conducted in a tertiary hospital from June to September 2024 in Shanghai, China.

### Participants

A purposive sampling was applied, aiming for a maximum variation sample to achieve sample diversity. The minimal sample size in the qualitative study is suggested as 12 to reach the data saturation [23]. Considering the largest attrition rate of longitudinal qualitative study was 50%, at least 24 older adults needed to be recruited to ensure data adequacy at each timepoint [22]. As the views of caregivers were collected to supplement the perspectives of the older adults, the sample size of caregivers was not considered.

This study focused on the changes in perceptions reporting the data of older adults with CAD and caregivers. The eligibility criteria for the older adults and caregivers were as follows:

Older adults with CAD: inclusion criteria were (1) aged 60 years old or over [24], (2) with a diagnosis of CAD, including the medical history of myocardial infarction, stable or unstable angina, undergoing percutaneous coronary intervention, or coronary artery bypass grafting [4, 25], (3) hospitalised because of CAD, (4) prescribed with at least one category of CAD medications (including renin-angiotensin system inhibitors, beta-blockers, lipid-lowering drugs, and antiplatelet agents) upon discharge; exclusion criteria were (1) had cognitive impairment (with Mini-cog score < 4) or a diagnosis of the mental condition(s), (2) having other communication barriers, such as aphasia, (3) planning to go to the nursing homes or long-term care facilities after discharge.

Informal caregivers: inclusion criteria were (1) aged 18 years old or above, (2) any family member, friend, or neighbour of the older adults with CAD, (3) self-identified or being identified by the older adults as their primary caregivers, (4) providing unpaid care for older adults; exclusion criteria were (1) having cognitive impairment (with Mini-cog score < 4) or a diagnosis of mental condition(s), (2) having other communication barriers, such as aphasia.

### Data collection

The participant enrolment and data collection were conducted by the first author (MQX) and a research assistant. MQX had undergone training in conducting qualitative studies and published papers about qualitative studies. She trained the research assistant (a registered nurse with a bachelor’s degree) in conducting interviews.

With the approval of the hospital, the interviewers checked the medical records in the cardiology department and identified the potential inpatients. Eligible older adults were invited to participate in individual semi-structured interviews conducted face-to-face in the wards half or one day before discharge, and face-to-face or online one month after discharge. The interviewers identified the eligible caregivers during the visiting time in the wards. The caregivers were interviewed once in the wards, meeting rooms, or online. The interview guide was predetermined based on literature review [26, 27] and the discussion of MQX and her supervisor (SHSL) (Supplementary 2).

The interviewers took field notes and used audio recordings for the interviews. Overall, 59 interviews were conducted, among which 21 older adults completed two interviews. One older adult only completed the interview one month after discharge. Six older adults dropped out in the follow-up because of the lack of interest (*n* = 2), lack of time (*n* = 2), feeling sick (*n* = 1), and loss of contact (*n* = 1). Interviews lasted for 26.0 min on average. The attrition rate is 21.4%. The caregivers were interviewed before hospital discharge (*n* = 8) or one month after discharge (*n* = 2), lasting for 26.2 min on average.

### Data analysis

Inductive approach was used in data analysis. We followed the six steps described in reflexive thematic analysis [28], focusing on the reflexivity for cross-sectional analysis, based on the recommendation of Braun and Clark on Big Q [29, 30]. Framework analysis was applied in longitudinal analysis [31]. The software MAXQDA 2020 was used in data encoding.

At first, MQX and the research assistant transcribed the recordings and cross-checked the accuracy. Interviews with doubts (*n* = 3) were taken back to the participants for verification. MQX read the transcripts repeatedly to get the initial idea. After getting familiar with the transcripts, she performed the open coding by the “comment” function of Microsoft Word 2018. The codes were grouped to generate potential themes together. Then, themes and sub-themes derived from the interviews of each participant before and after discharge were listed in a table to detect changes overtime. The changes in each theme of each participant were combined in another table for the longitudinal qualitative study. After reviewing the themes, MQX defined each theme and illustrated them with selected quotes. Finally, the codes, sub-themes, and themes were reviewed by SHSL. Disagreements were solved by discussion of MQX and SHSL, until a consensus was made. All data analysis was completed in Chinese and translated into English by MQX, who is fluent in English and Chinese. The themes and sub-themes were not taken back to the participants for checking.

### Reflexive statement

MQX is a registered nurse and a PhD student in nursing. She received the bachelor’s and master’s degrees in China. In her master’s study, she completed a clinical internship caring for patients with cardiac disease in a hospital in China and conducted research projects exploring their self-management behaviours. The experience is beneficial for her to identify the challenges in medication management among older adults with CAD. Meanwhile, it may influence her interpretation towards the findings. To enhance reflexivity, MQX discussed with the research team about the findings to ensure that the themes were grounded in the perceptions of older adults with CAD rather than preconceived assumptions.

### Rigour

Rigour of the qualitative study was ensured following the criteria of credibility, transferability, dependability, and confirmability [32]. The views of different stakeholders supported the triangulation of the data. For verifying the unclear information in the interviews, three older adults were invited for the member-checking of the transcripts. Themes, subthemes and quotes were cross-checked by the corresponding author. The strategies above ensured the credibility of the qualitative study. Transferability of the qualitative study is supported by maximum variation sampling. Audio recordings and filed notes improved the dependability. Repeatedly reviewing the transcripts and codebooks and involving the corresponding author in the data analysis maintained the confirmability.

## Findings

### Characteristics of the participants

 A total of 38 participants were included in this study, including 28 older adults with CAD and 10 caregivers. The mean age of the older adults with CAD was 69.5 ± 4.9 years. The characteristics of the older adults and the caregivers were summarised in Tables 1 and 2, respectively.

**Table 1:** Demographic and clinical information of the older adults with CAD (*n* = 28)

	*N* (%)
Age (years), Mean ± SD	69.5 ± 4.9 (range: 60–82)
Gender	Male: 16 (57.1%), Female: 12 (42.9%)
Educational level	Informal education: 1 (3.6%) Primary school: 4 (14.3%) Secondary school: 14 (50.0%) Tertiary or above: 9 (32.1%)
Marital status	Married: 25 (89.3%) Separated/Widowed/Unmarried: 3 (10.7%)
Living arrangement	Living with family: 26 (92.9%) Living alone: 2 (7.1%)
History of CAD	Newly diagnosed: 11 (39.3%) Being diagnosed before current admission: 17 (60.7%) <5years: 7 ≥ 5years: 10
Treatments during the current admission	Coronary angiography: 28 (100%) PCI: 15 (53.6%) CABG: 1 (3.6%) IABP: 1 (3.6%)
Number of medications taken before admission	0: 2 (7.1%) 1–4: 15 (53.6%) ≥ 5: 11 (39.3%)
Number of medications taken after discharge	4: 1 (3.6%) 5–8: 23 (82.1%) ≥ 9: 4 (14.3%)
Types of medications taken after admission	Aspirin: 20 (71.4%) P2Y_12_ receptor antagonists: 26 (92.9%) Lipid lowering drugs (statins, cholesterol absorption inhibitors): 28 (100%) Beta-blockers: 14 (50.0%) ACE-inhibitors or ARBs: 9 (32.1%) Diuretics: 4 (14.3%)
Number of comorbidities, Mean ± SD	2.1 ± 1.5

*PCI *Percutaneous Coronary Intervention, *IABP *Intra-aortic Balloon Pump, *CAD *Coronary Artery Disease, *ACE *Angiotensin-Converting Enzyme, *ARBs *Angiotensin II Receptor Blockers, *SD *Standard Deviation

**Table 2:** Demographic and clinical information of caregivers (*n* = 10)

No.	Caregivers						Older adults with CAD (care recipient)	
	Age (years)	Gender	Educational level	Occupation	Relationship with the older adults with CAD	History of taking care of the older adults with CAD	Age (years)	History of CAD
1	67	Female	Secondary school	Retired	Daughter	5 years	89	20 years
2	46	Male	Master’s degree	Working full-time	Son	4 years	81	3 years
3	53	Female	Secondary school	Working full-time	Daughter	0.5 years	82	1 month
4	57	Male	Secondary school	Working full-time	Son	10 years	83	Newly diagnosed
5	72	Female	Secondary school	Retired	Wife	4 years	73	3.5 years
6	65	Female	Primary school	Retired	Daughter	4 years	91	3 years
7	51	Female	Tertiary or above	Working part-time	Daughter	4 years	84	4 years
8	47	Male	Tertiary or above	Working full-time	Son	Unclear	82	Newly diagnosed
9	72	Female	Secondary school	Retired	Wife	1.5 years	72	4 years
10	71	Female	Primary school	Working part-time	Wife	8 years	73	8 years

*CAD *Coronary Artery Disease

Two themes along with five sub-themes were identified from 38 interviewees. The themes were “From compliance to engagement in medication management” and “Struggling in balancing the benefits and risks of medications”. The theme map with changes in perceptions of older adults was presented in Table 3.

**Table 3:** Theme map with changes in older adults’ perceptions of medications

Themes	Sub-themes	Codes		Flow of perceptions
		Before discharge (T1)	One-month post-discharge (T2)	
1. From compliance to engagement in medication management	1.1 Feeling compelled to accept to take the medications by disease	Perceiving the threat of disease	Taking medications for the fear of disease; The ambivalence of accepting the patient role	Disease perceptions remaining stable and fostering medication taking
		Perceiving less severity of CAD	Perceiving medications as unnecessary	
	1.2 Leaving health to the healthcare professionals	The authority of healthcare professionals, knowing little about medications is fine	Passive engagement in medication management: Active engagement in medication management	Role perceptions changing from obedience to independent administration
2. Struggling in balancing the benefits and risks of medications	2.1 Evolving understanding about the benefits of medications	Knowing little about CAD secondary prevention	Questioning the benefits of the medications	Dynamic perceptions about benefits and risks of medications (consistent/improved/decreased), leading to indecisiveness in maintaining adherence
		Misunderstanding the lipid lowering goal	Misunderstanding the lipid lowering goal; Improved understanding about the lipid lowering goal	
		Perceiving stable disease condition	Having confidence in adhering to medications	
	2.2 Predominant concerns about adverse drug events	Concerning about the side effects of CAD medications	Decreasing concerns about the side effects of CAD medications; Increasing concerns about the side effects of CAD medications	
		Concerns about adverse medication events caused by polypharmacy	Concerns about adverse medication events caused by polypharmacy	
		Inability to monitor the adverse medication events	Inability to monitor the adverse medication events; Enhanced ability to monitor the adverse medication events	
	2.3 Indecisiveness in maintaining adherence	Under pressure of balancing the benefits and risks of medications	Under pressure of balancing the benefits and risks of medications; Perceived risks of medications overweighing the benefits	
		Adopting self-adjusted strategies to balance the benefits and risks of medications	Adopting self-adjusted strategies to balance the benefits and risks of medications	

### Theme 1. From compliance to engagement in medication management

During hospitalisation, the older adults with CAD adhered to medication regimen driven by the threat of disease after the onset and the faith in healthcare professionals. The perception about threat of CAD kept consistent throughout the transition and fostered their medication-taking habit after discharge. The perceptions about their roles in medication taking shifted from being obedient patients during hospitalisation to independent administrators at home.

#### Sub-theme 1. Feeling compelled to accept to take the medications by disease

Perceived severity of CAD was identified as the major facilitators for adhering to medications, acknowledged by 19 older adults in the transition from hospital to home. Some of them were prescribed medications for CAD primary or secondary prevention before admission, but regarded them as unnecessary. After being informed about the severity of CAD based on the degree of coronary blockage of coronary angiography, they decided to take the medications during hospitalisation.

*“In this admission*,* they did a heart angiography for me. The doctor said that my heart’s blood flow was too slow*,* and I needed to take aspirin. In the past*,* I was afraid of taking it*,* but now I have to take it. It seems that I have no choice. I must listen to what the doctor says.” (15. Male*,* 73y*,* First time CAD _ T1)*

Three older adults took medication for better fulfilling the family responsibilities with well-being.

*“My major concern is family. I have to be responsible for my family*,* so I must take the medications. The doctor said*,* ‘If you don’t take them*,* your heart won’t work at all’. I have no choice. I must take them.” (17. Male*,* 69y*,* First time CAD _T1)*

The medication taking experience of others also influenced the acceptance among older adults by increasing their awareness about the severity of CAD and the importance of adhering to medications. Four older adults with CAD accepted taking CAD medications influenced by family history or their peers.

*“My mom also has CAD and has been on medication all along. Upon being discharged*,* the doctor informed me that I had to take medication for life. I have already accepted the lifelong medication.” (4. Male*,* 66y*,* First-time CAD_T2)*

*“Even if we don’t want to take the medications*,* we must take them. My peers are on medication. Anyone with a stent placed is on medication.” (23. Male*,* 75y*,* First-time CAD_T1)*

Understanding about disease severity was developed since their hospitalisation and maintained after discharge. Such understanding was regarded as helpful in maintaining their adherence to medications in the community. Twelve older adults reported that they continued to take the prescribed medications during the first month of discharge, as they understood their disease severity.

*“Before my heart attack*,* the doctor gave me statins*,* but I stopped taking them after just a few times. At that time*,* nothing happened*,* so I wasn’t worried. Now that I know I’ve had a heart attack*,* I realise I have to be more serious about medications.” (24. Male*,* 70y*,* First time CAD _ T2)*

After discharge, taking medications everyday was a “reminder” about their roles as patients. Given the nature of CAD, the symptoms of angina usually diminished after the surgeries. Medication taking reminded them about the chronicity and severity of CAD, which made them feel pain. Especially for those newly diagnosed with CAD, three of them had difficulty accepting the role of the patient and struggled in taking medications after discharge.

*“There are a lot of medications. I used to take no medication at all. I didn’t have any body issue. I even didn’t go for check-ups.” (17. Male*,* 69y*,* First time CAD_ T2)*

However, when older adults with CAD and their caregivers perceived little severity of CAD, they regarded medications as unnecessary and were unlikely to adhere to them after discharge.

*“I may not pay much attention to it (CAD medications). It seems that I take the medications*,* but without great attention. I haven’t found any issues in my heart now… I take it when I remember. Sometimes if I forget*,* I just skip it… Well*,* I think that not taking the medications doesn’t affect my heart.” (2. Male*,* 72y*,* CAD 4y*,* T2)*

#### Sub-theme 2. Leaving health to the healthcare professionals

The conception of the healthcare professionals as authorities was inherent in older adults with CAD and their caregivers. During hospitalisation, they were obedient patients, who chose to leave their health to the healthcare professionals without expressing their thoughts. Given the authority of healthcare professionals, 24 older adults unanimously regarded taking medications as simply following healthcare professionals’ instructions.

*“There’s nothing to worry about. Just listen to the doctor. What do I need to worry about? We don’t understand (medicine) and are not doctors.” (1. Male*,* 80y*,* CAD 15y_ T1)*

*“We are not healthcare professionals anyway. Just trust our doctors is enough. The best thing to do is to take the medications as prescribed and go for regular check-ups. There is no need to overthink or understand everything. Leaving the professional matters to the professionals.” (2. Son*,* 46y)*

As worrying the healthcare professionals being too busy or reluctant to address the queries, and concerning about being criticised, older adults were unwilling to communicate with them about their queries towards CAD medications in the hospital.

*“Even if you talk with the doctors (regarding concerns about side effects)*,* you still have to take these medications. There’s no way around it. Talking about it doesn’t really change anything. You need to take these medications. Even if you express your concerns to the doctor*,* they don’t have any other good alternatives.” (5. Male*,* 71y*,* CAD 12y_T1)*

*“Big hospitals are like this. When you get an appointment with a doctor*,* you wait for a long time*,* but the consultation only lasts one or two minutes. The doctor doesn’t say much and just writes prescriptions. They won’t give you extra information.” (3. Male*,* 72y*,* CAD 3y_ T2)*

Under the traditional notion about the authority of the healthcare professionals, two older adults with CAD also felt that their relationships with the healthcare professionals were unequal. They were in a subordinate position and lacked the power for communication on equal terms.

*“There’s no objection*,* no concern … ordinary people are like laboratory rats. What could we be concerned about?” (19. Male*,* 68y*,* CAD 9m_T1)*

*“The nurses give you a general overview (about medications). That’s enough. Patients can’t expect too much. To put it bluntly*,* we know our position. People in higher positions can talk more.” (14. Male*,* 71y*,* First time CAD_T1)*

Older adults with CAD and their caregivers always regarded themselves as non-professionals in medication management, who were incapable of understanding medications. Five older adults thought learning about CAD medications was meaningless to them and following instructions would be enough, as they were not healthcare professionals.

*“I feel that trying to understand the reasons behind taking aspirin and clopidogrel doesn’t help me. What I need to focus on is how to take them correctly and not miss any doses. That’s what matters to me. I need to manage them well. For the rest*,* I don’t care.” (19. Male*,* 68y*,* CAD 9m_T1)*

Three older adults with CAD felt powerless to control life due to old age. They were unwilling to think much about their disease and medications. They left everything to fate, and went with the flow.

*“No concerns at all. I live day by day.” (16. Female*,* 72y*,* First-time CAD*,* T1)*

After discharge from hospital, the older adults changed to a new role of being an independent administrator, but the engagement was sometimes passive. An older adult and three caregivers stated they only needed to follow orders, since excessive knowledge, especially about the side effects, would make them stressed.

*“Even if they (the healthcare professionals) told me about side effects*,* I still need to take the medications. I prefer not to think about those worrying things. I believe it’s better to know less. Otherwise*,* it just makes me more anxious. As long as I feel that the medication is good for my body*,* I’m happy to take it.” (12. Female*,* 69y*,* CAD 9y_T2)*

*“Once you understand the side effects*,* you won’t dare to take it*,* right?” (8. Son*,* 47y)*

The mindset of leaving the health to the healthcare professionals made older adults with CAD undervalue the importance of understanding the medications. There was a lack of initiative to derive medication information from discharge summaries, leaflets, and medication instructions after discharge.

*“I decide how to take my medication. I didn’t keep the discharge summary*,* and I won’t read it. Forget it.” (7. Female*,* 67y*,* First-time CAD_T2)*

*“I just need to know what medications to take. As for their benefits*,* I don’t think I need to understand them. Some people like to read the medication instructions with all that long stuff*,* but I don’t think I need to read them. I just need to know how to take it. That’s enough.” (12. Female*,* 69y*,* CAD 9y_ T2)*

On the contrary, four older adults with CAD actively raised questions about their medication management. The active involvement of patients increased the mutual trust between them and the healthcare professionals and was helpful for solving the problems in their medication management at home.

*“If I don’t understand something*,* I just ask. Otherwise*,* what can I do?… If we don’t ask*,* how will doctors answer us?… We can’t always rely on others. We need to think of ways ourselves.” (11. Female*,* 63y*,* CAD 1y_T2)*

### Theme 2. Struggling in balancing the benefits and risks of medications

Older adults with CAD had dynamic understanding about the benefits and risks of CAD medications before and after discharge depending on their experience and health status, which may increase or decrease their level of medication adherence, or remain consistent. They kept balancing the benefits and risks of medications and were indecisive in maintaining adherence after discharge.

#### Sub-theme 1. Evolving understanding about the benefits of medications

Older adults newly diagnosed with CAD and their caregivers reported knowing little about CAD secondary prevention before and after discharge. They were less confident about benefiting from CAD medications. Four of them thought their future life of taking CAD medications was full of uncertainty. “Wait and see” was a common method adopted by them, when transiting from hospital to home.

*“Right now*,* I’m not worried. I just follow the doctor’s advice. Let’s try this treatment for a while and see how it goes. If there’s no improvement*,* then I’ll have concerns…… I just started to take the medications*,* so I don’t know how things will turn out. Let’s wait and see for a bit.” (4. Male*,* 66y*,* First time CAD_ T2)*

Three caregivers also reported knowing little about the medications. A caregiver planned to consult the healthcare professionals about the usage upon discharge.

*“I’m not sure about names and effects of medications. I haven’t dealt with them before……Now*,* I just give him (his father) medications provided by the nurses*,* following the instructions. Before going back home*,* I will clearly ask them about the dosage of medications and remind him about it.” (8. Son*,* 47y)*

Their limited understanding of medications resulted in miscommunication after discharge. When the cardiologist suggested stopping the antiplatelet medications, a caregiver discontinued all the CAD medications as having no idea about the specific medications the doctor referred.

*“He (The doctor) just said we needed to stop the antiplatelet medication. Which one is the antiplatelet medication? I don’t know.” (3. Daughter*,* 53y)*

Different from relieving symptoms, the benefits of CAD secondary prevention may not be observed by older adults through somatic awareness or instrumental measurements (for example, the sphygmomanometer) at home. The imperceptible effects increased their difficulties in understanding the benefits of CAD secondary prevention. Two of them stopped taking the antiplatelet medications due to being unable to understand the benefits for the heart at home.

*“I have very few platelets. Actually*,* I’m fine if I don’t take (Ticagrelor).” (24. Male*,* 70y*,* First time CAD _ T2)*

Older adults were confused about the lipid-lowering treatments for CAD secondary prevention, including the benefits of lowering lipids and the targeted lipid levels. In the first interviews, three of the interviewees discontinued the statins at home as they thought their lipid level was in the normal range.

*“Recently*,* I’ve been off statins for two or three weeks. I don’t understand much about medicine. I just know that if my cholesterol is high*,* especially low-density lipoprotein (LDL) cholesterol*,* I should take this medication. But during my annual health check*,* my LDL levels have always been normal.” (20. Female*,* 74y*,* CAD 12y_T1)*

After discharging from the hospital, an older adult still queried about the benefits of statins and the goal of lowering the blood lipid level. Another older adult changed from being nonadherent to adherent to statins, because of the improvement in understanding the benefits of lipid-lowering treatments for CAD secondary prevention.

*“He (The doctor) said statins lowered blood lipids. Then he said that if you didn’t take them*,* your blood lipids would thicken*,* which could lead to blockages (in the vessels).” (10. Female*,* 63y*,* CAD 2y_T2)*

Two older adults reported having confidence in continuing adhering to CAD medications after discharge, because of the stable coronary artery stenosis during hospitalisation. In addition, they acknowledged the severity of CAD, and regarded it as stable after treatment.

*“After undergoing cardiology angiography*,* the doctor mentioned that my medication control was quite good and advised me to continue. It solidified my adherence to the medications*,* including the methods and timings. As a result*,* I feel more confident.” (25. Female*,* 68y*,* CAD 9y*,* T2)*

On the other hand, another older adult questioned the benefits of CAD medications after discharge, due to the fast progression of coronary artery stenosis despite of having taken the medications for two years.

*“When I had my first stent placed*,* they found a 35% narrowing in the left coronary artery. After taking medication for two years*,* it suddenly worsened to 85%. If I were to reason backwards*,* what would happen if I didn’t take the medication?” (22. Male*,* 60y*,* CAD 2y_T2)*

There were also distrusts towards the effects of domestically produced generic medications. Two older adults preferred to take imported medications for CAD secondary prevention, which were perceived as more effective than genetic medications.

*“It’s like cars. Imported*,* joint venture*,* and domestic models may all look similar*,* but their performance can be quite different. Everyone understands it. Similarly*,* the efficacy of generic medicines might also be affected.” (22. Male*,* 60y*,* CAD 2y_T2)*

#### Sub-theme 2. Predominant concerns about adverse drug events

Throughout the transition from hospital to home, 24 out of 28 older adults expressed concerns about adverse drug events. Facing the new prescriptions, their concerns increased after discharge, including fears about the possible side-effects of certain CAD medications, possibilities of drug duplications and interactions caused by polypharmacy.

Negative views from family, friends and social media about side-effects of CAD medications were conveyed to them, especially for the statins and antiplatelet medications. Besides, the traditional notion “All medications are somewhat toxic” (是药三分毒) was inherent among Chinese. Their concerns about CAD medications manifested in the following areas, including long-term medication diminishing the liver and kidney function, statins diminishing the liver function, and aspirin causing bleeding.

*“Actually*,* I don’t take statins. My friends said the side effects of this medicine were really severe*,* so I decided not to take it.” (27. Male*,* 69y*,* CAD 18y_T1)*

*“They (Others) said that taking too much aspirin made the blood vessels fragile.” (28. Female*,* 68y*,* First-time CAD_T2)*

Similarly, caregivers also highlighted the concerns about adverse drug events before staring the CAD medications.

*“All medications are somewhat toxic. There’s no way around it.” (8. Son*,* 47y)*

Notably, the percentage of older adults with polypharmacy (taking five medications or over) increased from 39.3% to 96.4% after discharge. Polypharmacy further increased their concerns over the safety of taking medications. Older adults with CAD had an average of 2.1 ± 1.5 comorbidities, such as diabetes, hypertension, liver and kidney dysfunction. Thirteen out of 28 of them worried about drug duplication and interactions, which would harm the liver and kidney function.

*“I keep wondering if taking all these medications together will cause side effects or if they will interact with each other. Some are for diabetes*,* some for heart disease. Can they be taken together? Will there be any side effects?” (10. Female*,* 63y*,* CAD 2y_T2)*

Though concerning about adverse drug events related to CAD medications and polypharmacy, many older adults and caregivers could not monitor them accurately. After discharge, two older adults reported feeling confused when experiencing some potential side effects of CAD medications and knew little about how to address them.

*“I took them (aspirin and tegretol) for two weeks. It seems there are bruises on my body. I can’t figure out why.” (17. Male*,* 69y*,* First-time CAD_ T2)*

Except routine check-ups, ten older adults with CAD reported seldom monitoring the biochemical indicators or blood pressure after taking medication. Five of them believed there was no need to monitor them, as long as they were feeling well. A caregiver reported she always helped her husband in getting the prescriptions, while omitting the importance of monitoring as her husband had no symptoms.

*“I get my blood tested every seven to eight months*,* not every time. Sometimes I feel well*,* so I skip it. It’s hard to say. Sometimes I do it*,* sometimes I don’t.” (9. Male*,* 62y*,* CAD 10y_T1)*

*“I kept helping him get prescriptions…… At first*,* after the stent was put in*,* he had check-ups every six months*,* then once a year. Later on*,* since we thought everything was fine*,* I just stopped taking him for check-ups.” (5. Wife*,* 72 y)*

Besides, there was inadequate support for older adults to get monitoring. Six participants found frequent monitoring troublesome. They faced difficulty commuting to hospitals, and experienced financial burdens for the tests. Two reported having difficulty interpreting their blood test results. Therefore, they could not receive timely and accurate feedback about adverse drug events after medication taking.

*“Doctors always suggest me to get blood tests every three months*,* and have an echocardiogram every six months. But I have no time for it. Nobody could take me to the hospital*,* and I can’t go by myself. So*,* I rarely go for tests. If you go for a full checkup*,* it costs over 1*,*000 RMB. We only have a retirement salary of 3*,*000 RMB every month. If you spend all of these on medications*,* there will be barely anything left for meals.” (6. Female*,* 72y*,* CAD 7y_T1)*

*“Well*,* I don’t have anyone to interpret it for me. I just read the blood test results myself. If the changes in various indicators are not significant*,* I would forget it. I won’t bother consulting them (the doctors).” (3. Male*,* 82y*,* CAD 3y_T2)*

After discharge, an older adult with CAD reported concerning less about the risks of medications after understanding the results of the blood tests for checking the liver and kidney functions.

*“I remember that my liver and kidney function were not affected. After being hospitalised*,* I had my liver function tested once*,* and it was normal.” (10. Female*,* 63y*,* CAD 2y_ T2)*

#### Sub-theme 3. Indecisiveness in maintaining adherence

The perceived benefits and concerns evolved among older adults with CAD throughout the transition from hospital to home. They struggled to balance the benefits and risks of CAD medications. Though acknowledging the benefits of CAD medications, the adverse drug events put them under great pressure and made them indecisive in maintaining adherence to medications.

*“You need to strike a balance. Excessive use of medications*,* like statins*,* might lead to damage for other organs. If you stop this medication and your cardiovascular issues or indicators worsen*,* you might face problems again. I believe it’s crucial to maintain this balance.” (21. Male*,* 71y*,* First-time CAD_T2)*

When returning home from hospital, the underlying perceptions about the medications resurfaced, as older adults changed from passively following the instructions of the healthcare professionals to taking control of the medication management. When beliefs about the risks of CAD medications outweighing the benefits, older adults discontinued CAD medications due to the fear of adverse drug events. Three older adults reported discontinuing CAD medications for the fear of risks when returning home. An older adult experienced bruises and became nonadherent to antiplatelet medications, when taking the new regimen at home. Two caregivers also discontinued CAD medications for the same reason.

*“I get scared looking at those bruises. They are not green*,* but red and purplish. They’re quite severe*,* quite frightening*,* so I reduce the medication on my own.” (12. Female*,* 69y*,* CAD 9y_ T2)*

*“Like the aspirins*,* I tried them myself first and felt uncomfortable. So*,* I didn’t give them to her (her mother). If I had the same disease with her*,* I would try them first. Just to be safe.” (1. Daughter*,* 67y)*

The indecisiveness could lead to nonadherence in the long run, because concerns increasingly outweighed perceived benefits over time. At one month of discharge, an older adult stated he would decrease the dosage of statins after the lipid level returned to the normal range to reduce the potential risks in future.

*“In the future*,* I would adjust it (statins) myself because it needs to be taken lifetime. It’s a lifelong medication…… So*,* if all my indicators were normal throughout a year*,* I would definitely reduce the dosage a bit to minimize the damage to my organs.” (21. Male*,* 71y*,* First-time CAD_T2)*

Polypharmacy could exaggerate the indecisiveness. Especially when new prescriptions were added, older adults may prioritise medications for other diseases to reduce the risks related to polypharmacy. An older adult discontinued the statins when she got flu, for the fear of drug interactions with the medications newly prescribed.

*“When I got other diseases*,* such as flu*,* I stopped taking the statins and take the medications for flu at first. I am afraid feeling uncomfortable in the stomach.” (12. Female*,* 69y*,* CAD 9y_ T1)*

Due to the conflicts in seeking the benefits in CAD secondary prevention and concerning about adverse drug events in taking medications, some older adults with CAD adopted self-adjusted approaches to reduce the risks of medications and retain their benefits, including adjusting the dosing time and taking health supplements. Three of them took medications (for example, aspirin) after meals, to reduce the risks of stomach discomfort. Five of them spaced out the intake of different medications (for example, 10 minutes, an hour), believing it would reduce the harm of taking multiple medications simultaneously.

*“Taking aspirin about half an hour after breakfast is safer. It is dangerous to take it on an empty stomach*,* as there could be risks if the time gap is too long. For example*,* if I forget to take aspirin after breakfast in the morning*,* and then remember an hour later*,* I wouldn’t dare to take it at that point.” (15. Male*,* 73y*,* First time CAD _ T2)*

*“I must take the medications. CAD persists. However*,* I still want to find a way to take them. So*,* I take them every ten minutes*,* trying not to take them all at once. If you take four or five pills at once*,* it will be harmful to your liver and kidney function. After all*,* these are chemical substances.” (9. Male*,* 62y*,* CAD 10y_T1)*

Because of the fear of the risks of western medications, some older adults with CAD took Chinese medicines and supplements. One of them wrongly believed that Chinese medicines and supplements could replace CAD medications.

*“Others said that red yeast rice(红曲)could replace statin medication. After taking it*,* three indicators*,* including cholesterol*,* low-density lipoprotein*,* and triglycerides*,* would drop quickly. If you take a larger dose consistently*,* you can expect a significant reduction in cholesterol levels in just three to four days.” (15. Male*,* 73y*,* First time CAD _ T2)*

## Discussion

This longitudinal qualitative study explored the changes in perceptions about medication management among older adults with CAD in the transition from hospital to home, involving two themes and five sub-themes. Three major patterns of changes in perceptions were identified, regarding the roles in medication management, disease, and medications. Older adults changed from simply following instructions by the healthcare professionals, who suppressed their doubts about the benefits and risks of medications during hospitalisation, to being independent administrators after discharge. They accepted to take medications after discharge, as perceiving consistent threat of CAD. Their understanding about the benefits and risks of CAD medications evolved in the transition from hospital to home. They struggled in balancing the benefits and concerns, and were indecisive in maintaining adherence.

We found that the older adults with CAD entrusted the authority of the healthcare professionals and suppressed their doubts during hospitalisation, who took medications obediently. Consistent with the existing evidence, the older adults trusted the healthcare professionals and were reluctant to ask questions about changing the regimens, because of the cohort effect of respecting the doctors and not daring to question the authority [33, 34]. In the traditional Chinese culture, the cultural value about “Mian Zi” emphasises the obedience, especially avoiding the open disagreements to the prescriptions of healthcare professionals [35, 36]. The communication between healthcare professionals and older adults could be improved to motivate the older adults to provide more feedback to understand more about their needs in medication management. Informing the patients (for example. assessing the understanding of medications, consultation, providing discharge medication lists), empowering them (for example. using teach-back techniques, solving the barriers in medication management) and involving their family members (for example, making admission medication list and interpreting the discharge summaries for patients) were potential strategies in improving medication communication during hospitalisation [37]. Notably, though becoming independent administrators after discharge, some older adults engaged in medication management passively, to avoid violating the health professionals and psychological burden. Continuous care has been suggested to improve the medication safety among the older adults after discharge. A systematic review about interventions in supporting medication management among the older adults in the transitional care found nine of 24 interventions were provided before and after discharge and six focused on the discharge period [38]. Goal setting and planning, feedback and monitoring, and instructions on behaviours were the most used techniques in improving medication management behaviours.

Older adults with CAD came to accept taking the medications after discharge triggered by the consistent threat of CAD. Existing studies indicated that the perceptions about identity, timeline, consequences, causes and control of disease was associated with medication adherence among patients with CAD [39, 40]. Patients with CAD tended to undervalue the consequences of CAD driven by symptoms and regarded it as curable after surgeries [41, 42]. Information provision about causes of CAD, consultation and interpreting the coronary angiography results would improve the perception about CAD severity in future interventions.

In the transition from hospital to home, the perception about medications evolved among the older adults with CAD, but concerns about adverse medication events were predominant. Different from patients with CAD from other countries [43], the conception of “All medications are somewhat toxic” was the specific perception among older adults in China. In China, under the cultural concept of “Yin Yang balance”, people prefer the traditional Chinese medicine to maintain the harmony of mind and body. Traditional Chinese medicine was perceived by them as natural with few side effects [35]. The preference for natural ingredients increased their concerns about the safety of western medications, who viewed them as treating the symptoms in surface in a short time [35]. After discharge, the concerns about medications increased because of polypharmacy, but the concerns decreased after understanding the ways to monitor the adverse medication events. Older adults with polypharmacy increased to over 90% after discharge. Polypharmacy was associated with medication nonadherence, because of various formulations and dosages, and increased concerns about adverse drug events [44, 45]. Providing information about the mild side effects (for example, slight bruises and dyspnea) with practical strategies to manage them at home and highlighting the severe side effects (for example, gastrointestinal haemorrhage and rhabdomyolysis) to seek medical help are also warranted in clinical practice. Older adults with CAD kept balancing the benefits and risks of medications before and after discharge. Decision aids (for example, quantitative pros and cons) work through the numerical format in weighting the benefits and risks of medications, consequently encouraging the patients to make decisions about adhering to medications [46, 47]. Moreover, designing interventions based on the cultural context and personal belief could optimise the interventions for improving medication adherence among Chinese. Regarding the fear of western medicine caused by the traditional notion, involving the family members, openly discussing the concerns, re-conceptualising the mild side-effects, and promoting the partner relationships between the healthcare professionals are potential strategies [48, 49].

This study indicated that perception about the threat of CAD as a determinant for medication adherence. Lack of understanding of benefits and concerns about side effects were another two critical barriers to medication adherence. In line with other studies, perceived threat of the disease, perceived benefits and risks of taking medication were crucial factors influencing medication adherence among patients with CAD [20, 43]. Coherent with the key findings of this study, Health Belief Model might be a potential theoretical framework in guiding the future interventions [50]. A study found that MA of the intervention group undergoing HBM-based motivation interviewing was 4.5 times than that of the control group. In this study, the perceptions about pulmonary tuberculosis and treatments were discussed to identify the barriers in MA and motivational interviewing was used in reducing the barriers [51].

In addition, we noticed that two out of ten caregivers suggested the older adults to discontinue CAD medications wrongly, due to a lack of understanding about CAD medications. As shown in literatures, caregivers involved voluntarily, who accompanied older adults with CAD and received medication education together [52, 53]. Some studies emphasised the important role of caregivers and acknowledged the support of caregivers as a critical component for designing interventions to improve medication safety among the older adults [54]. However, the independence of older adults was also valued by the caregivers, and it was suggested not to depend on the consistent and active engagement of caregivers in medication management [55]. For older adults with CAD at advanced age or with cognitive impairment, special educational sessions about medication management for their caregivers are needed. A systematic review synthesised the evidence about caregiver-based interventions. Pharmacist consultations, medication management trainings and education were found effective in improving the medication knowledge and self-efficacy in medication management of the caregivers [56].

## Limitation

Several limitations of this study should be acknowledged. First, purposive sampling was adopted to recruit the participants. Older adults with CAD were mainly with high education level and only two were 80 years old or over. All participants were recruited from Shanghai, which is one of the most developed cities in China. Their views may not represent patients in other regions. Second, 21 out of 28 older adults with CAD completed two interviews. Some potential changes in perception might be missed. Third, there was no quantitative measurement of medication adherence. Information in the interviews might be limited in understanding the degree of nonadherence. Forth, attention needs to be paid when interpreting the results, as the changes in medication management behaviours may be influenced not only by perceptions but also by contextual factors.

## Conclusion

Changes in perceptions about the roles in medication management, disease, and medications among older adults with CAD transitioning from hospital to home were observed in this study. Older adults shifted from compelling to take medications during hospitalisation to self-administration after discharge. Their perceptions about CAD were consistent throughout the transition and fostered their medication-taking behaviours after discharge. Their understanding about the benefits and risks of CAD medications evolved throughout the transition. They were indecisive in maintaining adherence. The views of caregivers were consistent with older adults. In future research and practice, components meeting their specific needs, such as patient-centred communication, cultural sensitivity, continued care, caregiver involvement, and psychological support in reducing concerns, are expected to improve their perceptions about medication management.

## Supplementary Information

Supplementary Material 1.

Supplementary Material 2.

## Data Availability

Data cannot be shared because participants are informed and agree to use their data in the current study.

## Abbreviations

CAD: Coronary artery disease
COREQ: Consolidated Criteria for Reporting Qualitative Research
