# Supplementary material for: Perceptions about medication management transitioning from hospital to home among older adults with coronary artery disease: a longitudinal qualitative study
Source: BMC Geriatr. 2026 May 12;26:910. doi: 10.1186/s12877-026-07610-8 (PMC13339563; doi:10.1186/s12877-026-07610-8)
Supplement: Supplementary file 1 — Supplementary Material 1. [file 12877_2026_7610_MOESM1_ESM.docx]

# **Supplementary 2. Interview guide**

**Questions for older adults with coronary artery disease (CAD)**

**Before discharge:**

1. Please tell me about the medications that you are currently taking at home.

(Prompts: CAD medications, OTC medications)

2. Please tell me how you usually manage your medications in your daily life.

(Prompts: getting prescription, storage, adherence, monitoring)

3. How would you view your role in managing medications?

(Prompts: dominant, active/passive)

4. What do you think are the challenges in managing the medications that you are currently taking at home?

(Prompts: getting prescription, storage, adherence, monitoring)

5. From 4, what strategies have you taken to address the challenges?

6. Could you name the CAD medications that you will take after discharge?

7. From 6. Do you have any concerns about these medications? What concerns you most regarding taking the medications?

(Prompts: effects, side-effects, drug interactions, drug dependence, cost)

8. What do you think will be the challenges you meet when managing CAD medications after discharge?

(Prompts: difficulty in getting prescriptions, forgetfulness, inadequate information, side-effects)

9. Did anyone explain the purpose of medications, side effects or usage to you? If yes, do you find it helpful?

10. What would you expect the healthcare professionals/caregivers to support you in medication taking after discharge from the hospital?

(Prompts: education, medication reminding, family support)

11. Do you have anything to share that we have not covered?

**After discharge:**

1. Please tell me about the CAD medications you are taking at home.

(Prompts: name, usage, effects, side-effects)

2. Please describe a normal day of you and how you take the CAD medications in your daily life.

(Prompts: getting up, having breakfast, having lunch, having dinner, going to bed)

3. Please tell me about how you manage the CAD medications at home.

(Prompts: getting prescription/storage, techniques, challenges/solutions)

4. Please tell me about the most memorable thing when you manage the CAD medications after discharge.

(Prompts: moving/challenging things, feelings, solutions)

5. How would you view your role in managing CAD medications?

(Prompts: dominant, active/passive)

6. Please describe the problems you concern when taking CAD medications. (Prompts: effects, safety, adherence, cost, solutions)

7. Have you ever make mistakes when taking CAD medications?

(Prompts: forgotten taken medications, missing the medications, taken the wrong medications)

8. From 7, what would you do to deal with the mistakes in medication taking?

(Prompts: skipping it, making it up in the next dose, making it up immediately)

9. What support have you ever received from others in medication management at home?

(Prompts: family/friends/healthcare professionals/internet, encouragement/ information/skill/reminding, before/after discharge)

10. What do you think of the support from others?

(Prompts: usefulness, necessity)

11. What would you expect the healthcare professionals/caregivers to support you after discharge?

(Prompts: education, medication reminding, family support)

12. What about your preference and needs for education programmes for CAD medications?

(Prompts: topics, delivery format, setting)

13. Do you have anything to share that we have not covered?

**Questions for the caregivers**

1. Please tell me about your role in supporting your family/friend (the older adult with CAD) in daily life.

(Prompts: care, support provided, relationship, burden)

2. How do you think of the differences of your roles in supporting your family/friend before and after their discharge?

(Prompts: dominant/support, time consumption, difficulty)

3. From 2, what do you think of the differences of your roles in supporting your family/friend in medication management after their discharge?

(Prompts: dominant/support, time consumption, difficulty)

4. How did you help your family/friend in medication management?

(Prompts: medication taking, storing, prescribing)

5. Please describe the challenges you had in supporting the medication management of your family/friend.

(Prompts: challenges, feelings, solutions)

6. How do you prepare for the home care after the discharge of your family/friend? (Prompts: medication management, nutrition, exercises)

7. What types of support would you expect to have to better support you in assisting family/friends in taking the medications?

(Prompts: education/psychological support, hospital/community)

8. Do you have anything to share that we have not covered?

**Questions for the healthcare professionals**

1. What do you think are the problems in medication self-management of older adults with CAD when transitioning from hospital to home?

(Prompts: getting prescriptions, taking medications, storing medications, concerns)

2. How do you help the older adults with CAD in medication management in your work?

(Prompts: information provision, skill training, reminding, WeChat group)

3. What do you think are the differences in medication management services you provide before and after discharge of the older adults with CAD?

(Prompts: topic, techniques, time)

4. What challenges have you met when managing CAD medications for older adults? (Prompts: time limitation, workforce)

5. What do you think of the roles of older adults with CAD in medication management?

(Prompts: dependent/independent, dominant)

6. What do you think of the roles of caregivers of older adults with CAD in medication management?

(Prompts: reminding, monitoring, psychological support)

7. What support do you need for better medication management of older adults with CAD?

(Prompts: patients, caregivers, hospital administrators, government administrators)

8. Do you have anything to share that we have not covered?
